# Upregulation of Soluble HLA-G in Chronic Left Ventricular Systolic Dysfunction

**DOI:** 10.1155/2016/8417190

**Published:** 2016-10-09

**Authors:** Line Lisbeth Olesen, Thomas Vauvert F. Hviid

**Affiliations:** ^1^Department of Cardiology, Zealand University Hospital (Roskilde), 10 Sygehusvej, 4000 Roskilde, Denmark; ^2^Department of Clinical Biochemistry, Centre for Immune Regulation and Reproductive Immunology (CIRRI), Zealand University Hospital (Roskilde) and Department of Clinical Medicine, University of Copenhagen, 10 Sygehusvej, 4000 Roskilde, Denmark

## Abstract

Left ventricular systolic dysfunction (LVSD) defined by ejection fraction (EF) <40% is common, serious but treatable, and correct diagnosis is the cornerstone of effective treatment. Biomarkers may help to diagnose LVSD and give insight into the pathophysiology. The immune system is activated in LVSD, and the immunomodulatory molecule human leukocyte antigen-G (HLA-G) may be involved. The primary aim was to measure soluble HLA-G (sHLA-G) in the blood in different stages of LVSD (<30% and 30–40%), in the midrange EF 40–50%, and in preserved EF ≥ 50% and to validate sHLA-G as a LVSD biomarker. The secondary aim was to examine associations between HLA-G gene polymorphisms influencing expression levels and LVSD. The 260 study participants were ≥75 years old, many with risk factors for heart disease or with known heart disease. Soluble HLA-G was significantly and uniformly higher in the groups with EF < 50% (<30, 30–40, and 40–50%) compared to EF > 50% (*p* < 0.0001). N-terminal fragment-pro-B-type natriuretic peptide (NT-proBNP) and uric acid values were inversely related to EF. According to Receiver Operating Characteristic (ROC) curves NT-proBNP outperformed both sHLA-G and uric acid as biomarkers of LVSD. Soluble HLA-G in blood plasma was elevated in LVSD regardless of EF. A novel finding was that a combined 14 bp ins-del/+3142 SNP HLA-G haplotype was associated with EF < 40%.

## 1. Introduction

The new European guidelines for heart failure define three groups based on ejection fraction (EF): a group with reduced EF < 40% (left ventricular systolic dysfunction (LVSD)); a group in the grey zone with EF in the midrange 40–49%; and a group with preserved EF ≥ 50%. It is concluded that patients in the grey zone probably have mild systolic dysfunction and the reason for creating a separate group is to stimulate research into characteristics, pathology, and treatment of this group of patients [1]. A problem related to the grey zone is an uncertain definition of LVSD, as shown and described in the echocardiographic study of the present study [2].

Left ventricular systolic dysfunction affects about 2% of the population in the western world, including many with unrecognized LVSD. It accumulates in the elderly population because LVSD is the final stage in most cardiac diseases, mostly caused by atherosclerosis in the coronary arteries [3]. The prognosis is grave, but treatment can delay progression and reduce morbidity and mortality. Screening for systolic heart failure in high risk populations should be considered because correct diagnosis is the cornerstone of effective treatment.

Echocardiography is the gold standard to diagnose LVSD, but access is limited, and referral to echocardiography requires a well-founded suspicion of LVSD [3]. Thus it is pivotal to look for new biomarkers, which might also give a better insight into the pathophysiology because LVSD is a complex disorder with hemodynamic, metabolic, neurohormonal, inflammatory, and immunological changes [4]. New biomarkers should be validated against established biomarkers.

B-type natriuretic peptide (or brain natriuretic peptide (BNP)) and N-terminal fragment-proBNP (NT-proBNP) are well established as diagnostic and prognostic biomarkers in LVSD, and combination with ECG might increase the specificity and the ability to screen for LVSD in high-risk populations [5, 6]. NT-proBNP synthesis is initiated by LVSD via neurohormonal activation and increased wall stress in the heart, and it is a hemodynamic cardiac marker. Uric acid is an old biomarker that may be ready for a renaissance [7].

Almost every disease and any injury to the body are accompanied by inflammation and activation of the immune system. The inflammatory system is complex and crucial for survival, but it is a double-edged sword. In LVSD, proliferation of monocytes and macrophages is observed. There is an increase in harmful oxygen-free radicals, primarily produced by xanthine oxidase; uric acid reflects xanthine oxidase activity. Proinflammatory cytokines with detrimental effects on myocardial function include tumour necrosis factor- (TNF-) *α*, interleukin-1, and interleukin-6 [4, 8–10]. These evoke a counterbalance reaction with increased production of anti-inflammatory interleukin-10 and HLA-G [11, 12].

This study aims to evaluate HLA-G as a new biomarker for LVSD. Human leukocyte antigen (HLA)-G is an HLA class Ib molecule with immunomodulatory, immunosuppressive, and tolerance-inducing functions [13]. It is well described in pregnancy protecting the fetus from an immune response from the mother. It is associated with a lower risk of rejection of a transplanted organ, [13–16], and, in cases of heart transplantations, myocardial expression of HLA-G has been significantly correlated with low risk of rejection [17]. In contrast, in pathological conditions, like infections and cancer, in which a vigorous and maintained immune response is desirable, the expression of HLA-G is detrimental. In cancer, it has deleterious escape-effects and the expression of HLA-G by the tumour cells seems to accelerate relapse [18]. The HLA-G protein has under normal conditions a very restricted expression pattern [13]. It is expressed during pregnancy by extravillous cytotrophoblast cells at the fetomaternal interface and is important for inducing maternal tolerance to the semiallogenic fetus [19, 20]. Furthermore, HLA-G is expressed by certain monocytes, T cells, and dendritic cells [21]. Four membrane-bound HLA-G isoforms and three soluble HLA-G isoforms generated by alternative splicing have been reported [13, 22]. Membrane-bound full-length HLA-G1 can also be cleaved from the cell surface by metalloproteinases [23].

A single published study has indicated that soluble HLA-G (sHLA-G) in plasma is upregulated in patients with systolic heart failure, compared to healthy controls, and independent of NYHA class, EF, and other biomarkers [24]. The study included only ten control subjects who were markedly younger than the participants in the present study. The current study compares sHLA-G with the state-of-the-art biomarker NT-proBNP and with uric acid, both independent biomarkers, in order to clarify if sHLA-G in blood plasma can be used as a biomarker for LVSD in a group of high-risk elderly persons. For the first time, two polymorphisms in the HLA-G gene are investigated in relation to EF. Several studies have indicated that these polymorphisms modulate HLA-G expression.

## 2. Materials and Methods

### 2.1. Patients and Samples

Individuals ≥ 75 years old from the general population and from a heart failure clinic, with heart disease risk factors or with former or present cardiac disease, especially LVSD, as well as healthy persons, were invited to participate. Two hundred and sixty subjects were included in the study. All participants provided written informed consent and the study was carried out in accordance with the ethical standards of the Declaration of Helsinki and was approved by the local ethics committee of Region Zealand and the Danish Data Protection Agency. Baseline characteristics of the study subjects are shown in Table 1.

While resting in supine position at room temperature all of the 260 subjects had a blood sample taken. Blood samples were obtained as EDTA plasma and heparin plasma samples, whole blood (EDTA tubes), and serum, with rapid flow from a large antecubital vein using standard venipuncture techniques. For the plasma samples, subsequently, centrifugation was performed to obtain platelet-poor plasma. The plasma supernatant was separated and the aliquot was transferred to cryotubes. All blood aliquots were stored at −80°C until analyzed.

Within the same hour a transthoracic echocardiography was performed by an experienced level 3 echocardiographer using General Electric Vingmed Vivid 7 or 9 and MJS probe 1.5–4.0 MHz and following guidelines from the Danish Society of Cardiology. Based on EF the study subjects were divided into four groups: (1) ≥50% (preserved EF, considered as normal), (2) midrange EF 40–50%, (3) LVSD with EF of 30–40%, and (4) LVSD with EF < 30%. Presence of moderate or severe valvular dysfunction was registered.

### 2.2. ELISA for Full-Length sHLA-G (The sHLA-G1 and HLA-G5 Isoforms)

The level of sHLA-G1/HLA-G5 molecules in blood plasma samples was determined by a commercially available sandwich enzyme immunoassay (ELISA) (EXBIO, Praha, Czech Republic) according to the manufacturer's instructions. This ELISA specifically detects sHLA-G1 and HLA-G5 in a *β*2-microglobulin- (*β*2m-) associated form. Samples were analyzed in duplicate on two independent assay plates, always with the same calibrators and positive and negative controls on each plate, and the first set of samples was also reanalyzed as the last samples to secure the reproducibility and precision of the assay. Blood plasma samples were diluted 1 + 3 with the provided Dilution Buffer (60 *μ*L samples to 180 *μ*L Dilution Buffer). Samples were thawed and mixed thoroughly and 100 *μ*L of diluted plasma were loaded in duplicate onto microtiter plates precoated with the monoclonal antibody MEM-G/9 (anti-HLA-G1/G5). The plates were then incubated overnight at 4°C (with no shaking). Following five washing steps with 350 *μ*L of the supplied washing buffer, 100 *μ*L of conjugate solution was added and the plates were incubated at room temperature (RT) for one hour. The conjugate solution consisted of monoclonal anti-human *β*2-microglobulin antibody labeled with horseradish peroxidase (HRP). After five additional washing steps, 100 *μ*L of substrate solution with tetramethylbenzidine (TMB) were added to the plate, and the plate incubated once more at RT for 25 min with no shaking. Finally, 100 *μ*L of acidic stop solution were added to the plates. The plates were then analyzed using a microplate reader. The assay was performed in a BEP2000 ELISA robot instrument (Siemens Healthcare Diagnostics, Germany).

### 2.3. Analysis of NT-proBNP and Uric Acid

NT-proBNP was measured on the Elecsys 2010 system (Roche Diagnostics). The assay is an electrochemiluminescent sandwich immunoassay that uses two polyclonal antibodies directed at residues 1–21 and 39–50 of the NT-proBNP molecule. The CV% of the assay is 3.2–2.4% from 20.7 to 585.5 pmol/L (175–4,962 ng/L) with an analytical range of 0.6–4138.6 pmol/L (5–35,000 ng/L). Plasma uric acid was measured on ARCHITECT ci8200 Integrated System (Abbott Diagnostics, North Chicago, IL, USA). One NT-proBNP test failed reducing the total number of study participants with a NT-proBNP test result to 259.

### 2.4. Genotyping of the 14 bp Insertion/Deletion Polymorphism (rs66554220) and the +3142 Single-Nucleotide Polymorphism (rs1063320) in the 3′-Untranslated Region of the HLA-G Gene

EDTA blood samples were carefully thawed and mixed thoroughly before processing. The DNA purification, using a Maxwell® 16 DNA Purification Kit, was performed in accordance with the manufacturer's instructions, and genomic DNA was stored at −20°C for further use. The real-time TaqMan PCR assay for genotyping of the HLA-G 14 bp insertion/deletion (ins/del) polymorphism in exon 8 (rs66554220) was performed using a LightCycler480 instrument (Roche Diagnostics, Switzerland) and performed as described by Djurisic et al. [25]. The genotyping of the +3142 SNP in the 3′UTR of the HLA-G gene (rs1063320) was performed as described by Bortolotti et al. [26].

### 2.5. Statistical Analysis

Specific* a priori* hypotheses were formulated. Each variable was tested for Gaussian (normal) distribution. In cases of a normal distribution, parametric tests were used (one-way ANOVA and unpaired *t*-test). Else, nonparametric tests were used (Kruskal-Wallis test, Mann–Whitney *U* test, and Jonckheere-Terpstra test). Receiver Operating Characteristic (ROC) curves were drawn for sHLA-G, proBNP, and uric acid. Charlson comorbidity index was calculated as described by Hall et al. [27]. Statistical analyses were made with the use of IBM SPSS version 22.0. Graphs were made in GraphPad Prism version 6 and IBM SPSS.

## 3. Results

### 3.1. Characteristics of the Study Group

Table 1 shows the baseline characteristics of the study group (EF < 40%) versus the control group (EF ≥ 40%). According to echocardiography there were 154 (59.2%) subjects with EF ≥ 50%, 106 (40.8%) subjects with EF < 50%, 45 with EF 40–50%, and 61 (23.5%) with EF < 40% (30 with EF 30–40% and 31 with EF < 30%); 55 subjects had mitral or aortic valvular dysfunction to some degree, no one had severe valvular disease, and 20 patients with valvular disease also had LVSD.

### 3.2. Soluble HLA-G Levels in Blood Plasma Are Associated with Heart Failure

**Figure 1:**
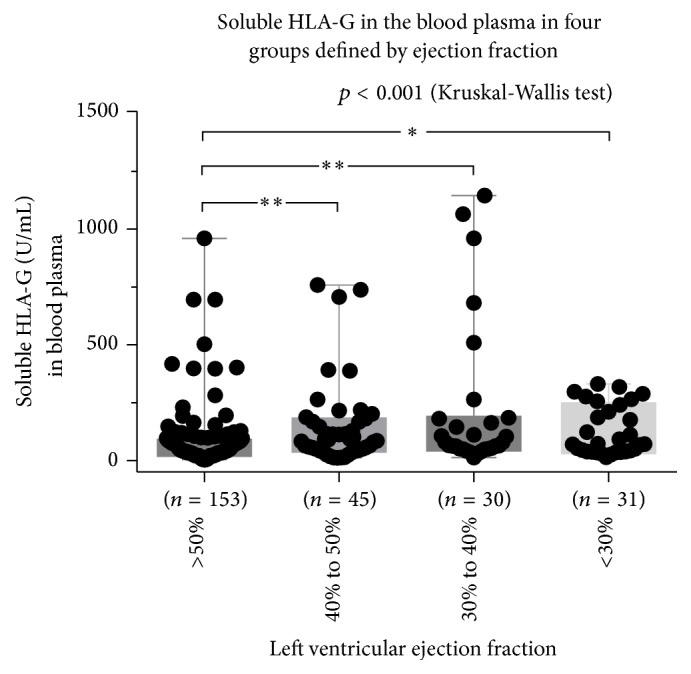
Soluble HLA-G in peripheral blood in relation to left ventricular ejection fraction (box and whiskers plot, min. to max., all points shown; *p* < 0.001, Kruskal-Wallis test; Dunn's multiple comparisons test, ^*∗*^ *p* < 0.05, ^*∗∗*^ *p* < 0.01) (one NT-proBNP test failed in the group with ejection fraction >50% reducing the number to 153).

**Figure 2:**
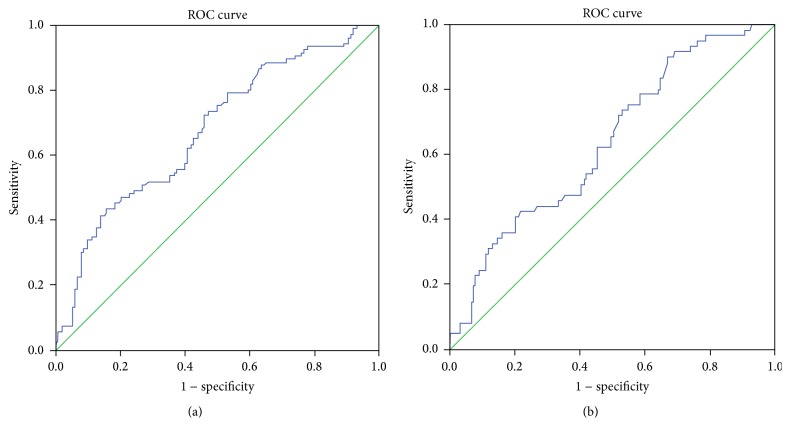
(a) Receiver Operating Characteristic (ROC) curve of soluble HLA-G with heart failure defined as ejection fraction <50%. Area under the curve is 0.676, *p* < 0.001. (b) Receiver Operating Characteristic (ROC) curve of soluble HLA-G with heart failure defined as ejection fraction <40%. Area under the curve is 0.639, *p* = 0.001.

**Figure 3 fig3:**
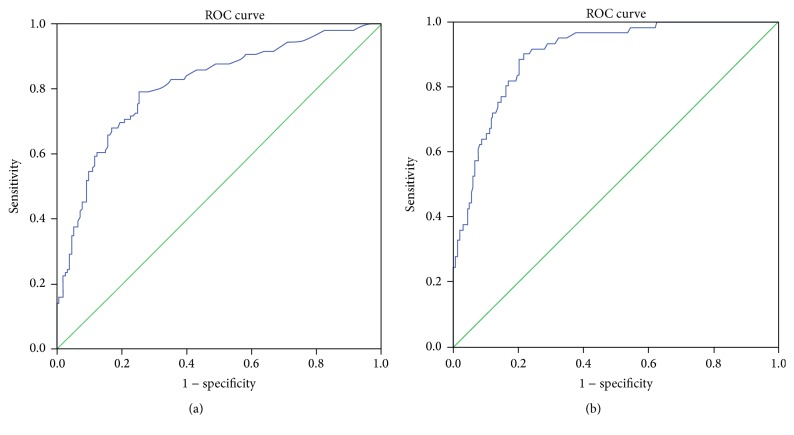
(a) Receiver Operating Characteristic (ROC) curve of NT-proBNP with heart failure defined as ejection fraction <50%. Area under the curve is 0.811, *p* < 0.001. (b) Receiver Operating Characteristic (ROC) curve of NT-proBNP with heart failure defined as ejection fraction <40%. Area under the curve is 0.902, *p* < 0.001.

**Figure 4:**
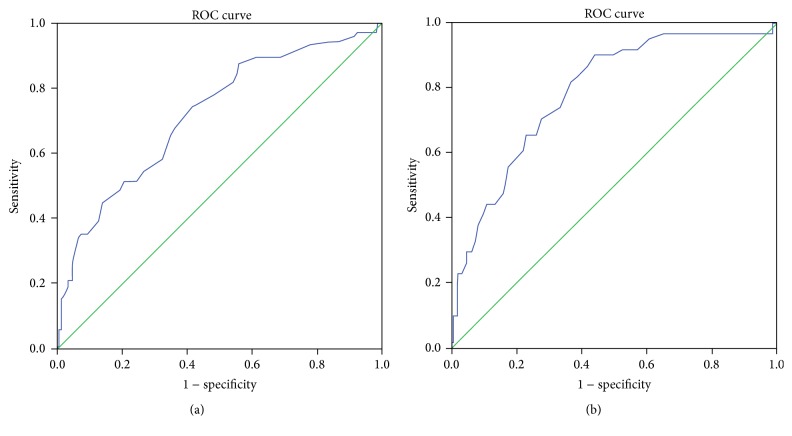
(a) Receiver Operating Characteristic (ROC) curve of uric acid with heart failure defined as ejection fraction <50%. Area under the curve is 0.721, *p* < 0.001. (b) Receiver Operating Characteristic (ROC) curve of uric acid with heart failure defined as ejection fraction <40%. Area under the curve is 0.788, *p* < 0.001.

Soluble HLA-G in the blood plasma was significantly and uniformly higher in the two LVSD groups with EF < 30% and EF 30–40% and in the midrange group with EF 40–50%, compared to the group with preserved EF ≥ 50% (*p* < 0.0001, Kruskal-Wallis test (Figure 1 and Table 2)). The values of NT-proBNP and uric acid were increased with decreasing EF (Table 2). Furthermore, Receiver Operating Characteristic (ROC) curves showed that NT-proBNP outperformed both sHLA-G and uric acid as a biomarker of LVSD (Figures 2 [Fig fig3]–4).

There was no correlation between sHLA-G and uric acid, neither in the whole population nor in the patients with LVSD (Spearman, *p* = 0.295, *n* = 256; *p* = 0.529, *n* = 105).

**Figure 5:**
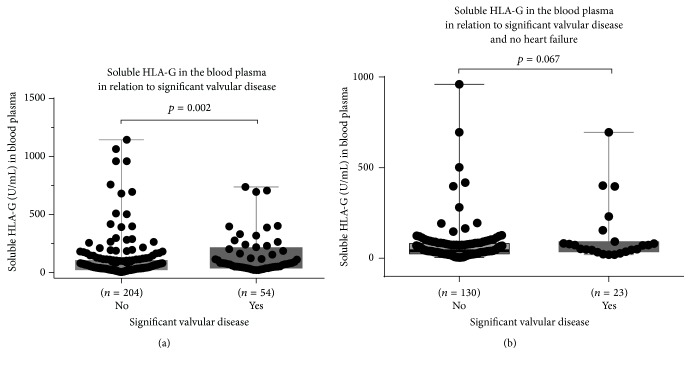
(a) Soluble HLA-G in peripheral blood in relation to significant valvular heart disease (box and whiskers plot, min. to max., all points shown; *p* = 0.002, Mann–Whitney test). (b) Soluble HLA-G in peripheral blood in relation to significant valvular heart disease in patients without heart failure (EF > 50%) (box and whiskers plot, min. to max., all points shown; *p* = 0.067, Mann–Whitney test).

There was a significant higher level of sHLA-G in cases of significant valvular heart disease for the whole study population of 260 subjects (*p* = 0.002, Mann–Whitney test Figure 5(a)). However, there was only a trend for the fraction of the population without LVSD (*p* = 0.067; Figure 5(b)).

### 3.3. HLA-G Gene Polymorphisms and Association with Risk of Heart Failure

There were no significant differences in the distributions of the 14 bp ins/del genotypes between the different EF groups (Table 3; *p* = 0.82, Chi-Square test). However, there was a tendency, when the group with EF > 40% was compared with the group with EF < 40% (*p* = 0.10, Chi-Square test). In the group with EF > 40%, the frequency of the Ins 14 bp/Ins 14 bp genotype was 19.1% and only 8.2% in the group with EF < 40% (Table 3).

This difference was statistically significant, when the combined haplotype of the two HLA-G gene polymorphisms, the 14 bp ins/del and the +3142 SNP, was analyzed (Table 4; *p* = 0.033, Chi-Square test). The combined genotype of the haplotype InsG/InsG was more frequent among subjects with EF > 40% than among patients with EF < 40%; the opposite was observed for the DelG/InsG combination of haplotypes.

In a separate analysis of the +3142 HLA-G SNP alone, no differences were observed in the distributions of the three genotypes between the group with EF < 40% and the group with EF > 40%. For the group with EF > 40%, the frequencies were C/C (32.2%), C/G (43.7%), and G/G (24.1%). For the group with EF < 40%, they were C/C (32.8%), C/G (44.3%), and G/G (23.0%).

## 4. Discussion

This study shows that sHLA-G is increased in patients with EF < 40 compared to patients with EF ≥ 40%. There was no difference in the concentration of sHLA-G in LVSD patients with EF < 30% and with EF between 30 and 40%. Thus, sHLA-G in the blood plasma does not indicate the severity of LVSD, and, in accordance with the study by Almasood et al., we conclude that sHLA-G is a very sensitive LVSD biomarker [8, 9, 24]. The assay used in the current study detects both soluble HLA-G5 and soluble HLA-G1 associated with *β*2-microglobulin. The source of sHLA-G in the blood from men and nonpregnant women is not well established but is probably derived from immune cells [21]. It can be speculated that the raise in sHLA-G associated with LVSD might originate from activated immune cells or from the heart.

Neither sHLA-G nor serum uric acid is comparable with NT-proBNP as LVSD-biomarker, primarily due to a poor specificity [5]. Soluble HLA-G is influenced by many other conditions, for example, cancer and autoimmune disease, which may confound the analysis and the results and reduce the suitability of sHLA-G as a biomarker for LVSD.

Specificity may be increased by genotyping for the combined haplotypes of the two tested HLA-G gene polymorphisms. An interesting novel finding in the current study was that the distribution of the combined haplotypes of the two tested HLA-G gene polymorphisms, the 14 bp ins/del and the +3142 SNP in the 3′UTR of the gene, was statistically significant between the subjects with EF > 40% and patients with EF < 40% (Table 4; *p* = 0.033). InsG/InsG was more frequent among subjects with EF > 40% than patients with EF < 40%; the opposite was observed for DelG/InsG. Interestingly, in healthy blood donors, the Ins 14 bp/Ins 14 bp HLA-G genotype has been significantly associated with low sHLA-G levels in the blood in several studies [28, 29]. Furthermore, the DelG/InsG combination has also been associated with a higher level of sHLA-G than the InsG/InsG combination, for example, in patients with multiple sclerosis; however, the DelC/DelC combination showed the highest concentrations of sHLA-G in the same study [30]. This is supported by the observations in the current study, which could indicate that specific HLA-G gene polymorphisms or haplotypes might influence the sHLA-G level in the blood and thereby the individual sHLA-G response in specific patients with systolic heart failure. It is not known whether this is due to genetic or epigenetic factors or if it is an adaptive mechanism triggered by the inflammatory process [19, 20].

There are certain limitations to the present study. One limitation of the current study is that the number of study participants is rather small; however, it is still the largest study until now regarding HLA-G in subjects with and without systolic heart failure. The study reflects clinical practice. The diagnosis of heart failure is uncertain for the small group with EF in the grey zone of 40–50%, but echocardiography is the standard diagnostic test and in this study performed by the most qualified [1, 2]. Important confounding factors are morbidities other than systolic heart failure, and these are common and inevitable (Table 1).

One of the reactions of the body to injury is inflammation represented in this study by serum uric acid, which might trigger an immunologic response represented in this study by sHLA-G [10]. Both are biomarkers of LVSD, and thus inflammation and immune modulation seem to be involved in LVSD. This is in accordance with accumulating evidence that systemic and persistent inflammatory disorders predispose to cardiovascular diseases. This is the case in gout, rheumatoid arthritis, psoriasis, inflammatory bowel disease, lupus erythematosus, sclerosis disseminatus, and other autoimmune diseases and in chronic infections and cancers [4, 5, 18, 31]. It is also observed in conditions associated with long-lasting low grade inflammation and endothelial dysfunctions like atherosclerosis, diabetes mellitus, the metabolic syndrome, venous thromboembolism, smoking, and affective disorders and in chronic heart failure [4, 12]. Upregulation of HLA-G is present in most of these disorders, which nevertheless are dominated by inflammation [18]. Out-of-balance inflammation with persistent rise in inflammatory cytokines seems to be the common denominator for many potentially coherent diseases and disorders, and it acts self-reinforcing in a complex vicious circle [4]. The inflammatory triggers and mediators are poorly understood, but they promote and regulate the inflammatory cascade that predisposes to, for example, atherosclerosis and LVSD [31].

HLA-G is elevated in many conditions, and thus there is a lack of diagnostic precision and specificity, which may obscure evaluation of the significance of HLA-G as a biomarker of LVSD in a multimorbid ageing population like the one in the current study. Furthermore, it cannot be determined from the current study, which role sHLA-G might have in the pathogenesis and the clinical course and prognosis of LVSD and whether it is a simple marker or participate in LVSD.

Likewise, the pathophysiologic effect of uric acid in LVSD is unknown. Serum uric acid has a negative correlation with EF [8, 9]. It is an independent risk factor for LVSD, but it is not known if it is cause, consequence, or simply an epiphenomenon. The serum uric acid concentration is increased in patients with chronic LVSD, probably due to both reduced renal excretion and augmented production [8, 9]. Low-sodium diet, diuretics, and insulin resistance may increase reabsorption of uric acid. Cardiac and renal disorders are related and as cardiac function deteriorates with falling cardiac output, the glomerular filtration rate (GFR) falls, which leads to a reduction in renal uric acid excretion [10]. At the same time the inflammatory process associated with the chronic diseases accelerates, which contributes to an increase in serum uric acid, TNF, interleukin-1 and interleukin-6, and other cytokines and in sHLA-G [7]. The simultaneous elevation of uric acid and sHLA-G might represent different aspects of the same process, acting in negative feedback as proinflammatory and anti-inflammatory markers.

In LVSD there is an imbalance between proinflammatory and anti-inflammatory cytokines [32]. The clinical significance remains to be determined. There are indications that the prognosis may be improved by restraining the inflammatory process, and an increase in EF in LVSD has been observed following treatment with, for example, thalidomide, pentoxifylline, intravenous immunoglobulin, glucocorticoid, colchicines, methotrexate, biological agents, interleukin-10, influenza vaccination, HIV-therapy, antidiabetic sodium glucose cotransporters, and antidepressant drugs [4, 12, 32, 33]. However, no improvement was observed in a variety of antibiotic trials and in studies antagonizing TNF in patients with LVSD, and there have been mixed results in studies of the use of allopurinol, which inhibits xanthine oxidase [4, 8, 11, 12, 31]. In the future, therapies directed at downregulating or inhibiting inflammation may reduce atherosclerosis and its complications including heart failure [33]. Further studies are needed to elucidate the role of HLA-G in this scenario.

## 5. Conclusion

For the first time, it was shown in the current study that a combined haplotype (DelG/InsG) of two HLA-G gene polymorphisms, the 14 bp ins/del and the +3142 SNP, were more frequent among patients with EF < 40% than among subjects with EF ≥ 40%. The opposite was observed for the combined haplotype InsG/InsG. This probably influences the sHLA-G level in the blood, and this study also showed that sHLA-G was increased in patients with EF < 40 compared to patients with EF ≥ 40%.

## Figures and Tables

**Table 1:** Baseline characteristics of the study population (LVSD, EF ≤ 40%) compared to the control group (EF > 40%).

Characteristics	Ejection fraction ≤ 40%		Ejection fraction > 40%		All together	
	LVSD: yes		LVSD: no		Number of individuals (*n* = 260)	Percentage
	Number of individuals (*n* = 61)	Percentage	Number of individuals (*n* = 199)	Percentage		
Age (years)	81		80		80 (75–92)	
Gender						
Females	14	23	119	60	134	52
Males	47	77	80	40	126	48
Body mass index (BMI) (kg/m^2^)	26.1		25.4		25.6	
Smoking						
Present	8	13	18	9	26	10
Ex-smoker	35	57	95	48	130	50
Never smoker	18	30	86	43	104	40
History						
Angina pectoris	25	41	52	26	77	30
Acute Myocardial Infarction (AMI)	31	51	24	12	55	21
Percutaneous Coronary Intervention (PCI)	12	20	27	14	39	15
Coronary Artery Bypass Grafting (CABG)	15	25	15	8	30	12
Dilated cardiomyopathy	10	16	5	3	15	6
Valvular disorder	17	29	12	6	29	11
Valvular substitution	6	10	4	2	10	4
Arrhythmias	45	74	47	24	92	35
Atrial fibrillation P-P-P	38	62	37	19	75	29
Pacemaker	25	41	4	2	29	11
Hypertension	41	67	132	66	173	67
Diabetes mellitus	15	25	22	11	37	14
Hypercholesterolaemia	41	67	103	52	144	55
Thyroidal disease	6	10	25	13	31	12
Cerebrovascular disease	14	23	29	15	43	17
Peripheral arterial disease	10	16	13	7	23	9
Lung disease	27	44	40	20	67	26
Gastrointestinal disorder	33	54	91	46	124	48
Renal disorder (moderate-severe)	10	16	11	6	21	8
Musculoskeletal disease	41	67	119	60	160	62
Autoimmune disease	4	7	16	8	20	8
Anemia	10	16	11	6	21	8
Cancer	14	23	54	27	68	26
Charlson comorbidity index	3.7		1.5		2.0	
Corrected for age	7.3		4.9		5.5	
QRS-duration	138		94		104	
Medical treatment						
Thrombocyte inhibitor	36	59	82	41	118	45
AK	23	38	30	15	53	20
Statin	35	57	74	37	109	42
Diuretics	46	75	74	37	120	46
Diuretics (Centyl)	10		49			
Diuretics (Furosemide)	32		23			
Aldosterone-antagonist	18	30	8	4	26	10
ACE-inhibitor	34	56	42	21	76	29
ATII	14	23	32	16	46	18
Beta-blockers	49	80	61	31	110	42
Clinical chemistry	Mean		Mean		Mean	
Hemoglobin (mmol/L)	8.1		8.2		8.2	
Potassium (mmol/L)	4.0		3.8		3.9	
Sodium (mmol/L)	139		139		139	
Creatinine (*µ*mol/L)	116		84		92	
GFR	55		69		66	
C-reactive protein (CRP) (mg/L)	5.0		4.5		4.6	
Thyroid stimulating hormone (TSH) (mU/L)	1.3		1.6		1.5	
Albumin (g/L)	39		41		41	
Alanine aminotransferase (ALAT) (U/L)	20		21		21	
Uric acid (mmol/L)	0.49		0.35		0.38	
Glucose (mmol/L)	6.6		5.9		6.1	
NT-proBNP (pmol/L)	268		50		101	
Soluble HLA-G (U/mL)	71 (12–1144) (median, range)		52 (4–960) (median, range)		61 (4–1144) (median, range)	

**Table 2:** The distribution of soluble HLA-G (U/mL), NT-proBNP (pmol/L), and uric acid (mmol/L) according to the left ventricular ejection fraction (median, range).

Parameter	All study population	*n*	Left ventricular ejection fraction			
			>50%	40% to 50%	30% to 40%	<30%
Soluble HLA-G (U/mL)	61 (4–1144)	259	44 (4–960)	90 (11–758)	71 (12–1144)	71 (14–331)
NT-proBNP (pmol/L)	35 (4–1620)	259	24 (4–347)	41 (7–273)	134 (21–389)	307 (69–1620)
Uric acid (mmol/L)	0.37 (0.18–0.92)	257	0.34 (0.18–0.87)	0.37 (0.20–0.68)	0.44 (0.19–0.80)	0.44 (0.19–0.92)

**Table 3:** Distribution of genotypes of the 14 bp insertion/deletion polymorphism in the 3′-untranslated region of the HLA-G gene in relation to left ventricular ejection fraction.

Ejection fraction	Del 14 bp/Del 14 bp	Del 14 bp/Ins 14 bp	Ins 14 bp/Ins 14 bp	Total
>50%^a^	61 (39.6%)	66 (42.9%)	27 (17.5%)	154 (100.0%)
40% to 50%	17 (37.8%)	17 (37.8%)	11 (24.4%)	45 (100.0%)
30% to 40%	12 (40.0%)	16 (53.3%)	2 (6.7%)	30 (100.0%)
<30%	12 (38.7%)	16 (51.6%)	3 (9.7%)	31 (100.0%)

>40%^b^	78 (39.2%)	83 (41.7%)	38 (19.1%)	199 (100.0%)
<40%^b^	24 (39.3%)	32 (52.5%)	5 (8.2%)	61 (100.0%)

^a^ *p* = 0.82 (Chi-Square test, 0.40, df = 2); ^b^ *p* = 0.10 (Chi-Square test, 4.57, df = 2).

**Table 4:** Distribution of haplotypes of the 14 bp insertion/deletion polymorphism (Del/Ins) and the +3142 SNP (C/G) in the 3′-untranslated region of the HLA-G gene in relation to left ventricular ejection fraction.

Ejection fraction	DelC/DelC	DelC/DelG	DelC/InsC	DelC/InsG	DelG/DelG	DelG/InsG	InsG/InsG	Total
>40%^a^	62 (31.2%)	15 (7.5%)	1 (0.5%)	74 (37.2%)	1 (0.5%)	8 (4.0%)	38 (19.1%)	199 (100.0%)
<40%^a^	20 (32.8%)	4 (6.6%)	0 (0.0%)	23 (37.7%)	0 (0.0%)	9 (14.8%)	5 (8.2%)	61 (100.0%)

^a^ *p* = 0.033 (Chi-Square test, 10.50, df = 4), for the Chi-Square test DelC/InsG was added to the DelC/DelC group and DelG/DelG was added to the DelG/InsG group.
